# Osteogenic potential of mesenchymal stem cells from rat mandible to regenerate critical sized calvarial defect

**DOI:** 10.1177/2041731419830427

**Published:** 2019-03-12

**Authors:** Dong Joon Lee, Jane Kwon, Luke Current, Kun Yoon, Rahim Zalal, Xiangxiang Hu, Peng Xue, Ching-Chang Ko

**Affiliations:** 1 Oral and Craniofacial Health Sciences Research and North Carolina Oral Health Institute, School of Dentistry, The University of North Carolina at Chapel Hill, Chapel Hill, NC, USA; 2 Department of Orthodontics, School of Dentistry, The University of North Carolina at Chapel Hill, Chapel Hill, NC, USA

**Keywords:** Mesenchymal stem cells from mandible, critical sized defect, mandible, craniofacial bone tissue engineering

## Abstract

Although bone marrow–derived mesenchymal stem cells (MSCs) have been extensively explored in bone tissue engineering, only few studies using mesenchymal stem cells from mandible (M-MSCs) have been reported. However, mesenchymal stem cells from mandible have the potential to be as effective as femur-derived mesenchymal stem cells (F-MSCs) in regenerating bone, especially in the orofacial regions, which share embryonic origin, proximity, and accessibility. M-MSCs were isolated and characterized using mesenchymal stem cell–specific markers, colony forming assay, and multi-potential differentiation. *In vitro* osteogenic potential, including proliferation, osteogenic gene expression, alkaline phosphatase activity, and mineralization, was examined and compared. Furthermore, *in vivo* bone formations of F-MSCs and M-MSCs in rat critical sized defect were evaluated using microCT and histology. M-MSCs from rat could be successfully isolated and expanded while preserving their MSC’s characteristics. M-MSCs demonstrated a comparable proliferation and mineralization potentials and *in vivo* bone formation as F-MSCs. M-MSCs is a promising cell source candidate for craniofacial bone tissue engineering.

## Introduction

Mesenchymal stem cells (MSCs) have been a prominent cell source in therapeutic strategies for replacing damaged or diseased bone in the field of bone tissue engineering for many years.^1–3^ These MSCs have primarily been sourced from bone marrow of the femur, tibia, and pelvic bone. However, harvesting bone marrow is a technically demanding procedure associated with long surgical times, donor site morbidity accompanied by the need for a second surgical site, and extended postsurgical pain management. MSCs can also be isolated from various orofacial tissues, including dental pulp, apical papilla, periodontal ligament, and stem cells from human exfoliated deciduous teeth.^4,5^ An increasing number of studies have reported the osteogenic potential of orofacial MSCs as an alternative cell source for bone regeneration. In most of the studies, MSCs derived from these oral tissues have been compared to their MSCs counterparts derived from bone marrows of non-orofacial regions and have been shown to have comparable multipotent differential capacity by MSC-specific marker expression and in vivo bone formation.^6–9^

Although there is a rising interest in utilizing orofacial MSCs as a cell source for dental and craniofacial bone regeneration, very few studies exist that utilize MSCs harvested from the bone marrow of the mandible (M-MSCs). Historically, M-MSCs have been isolated from mice, specifically the bone marrow of the mandible, using a syringe. The extracted bone marrow was then enzymatically digested to yield MSCs.^10^ However, this method involves a high risk of contamination with other types of cells in the culture system. In addition, the brittleness and exceedingly small size of the mandible in mice make it difficult to locate and isolate bone marrow and result in high rate of failure. The use of large animals could negate this limitation; however, using a large animal model is very costly in terms of housing, surgical procedures, and data analysis. Also, it occasionally involves ethical considerations.

M-MSCs sourced from the mandible can be one of the aforementioned alternative sources for MSCs and show potential in being useful in repairing defects in the orofacial region as they share embryonic origin, proximity, and accessibility. Neural crest-derived bones differ from appendicular bones in developmental origin, mode of bone formation, and pathological bone resorption. A few studies reported utilizing MSCs isolated from murine, canine, porcine, and human, but the reason for the differences in osteogenic potential between M-MSCs and MSCs derived from non-orofacial regions, specifically the femur (F-MSCs), is not clearly understood.^11–13^ Regarding to murine M-MSCs, Yamaza et al.^14^ reported that murine M-MSCs are distinct from F-MSCs with respect to their osteogenic potential and can interplay with T-lymphocytes for their immunomodulatory characteristics. These researches gave an insight to explore a new source of MSCs existing in the bone marrow in the orofacial region and a possible method of using allogeneic MSC transplantation for bone regeneration. We surmise that the difference stems from the fact that bone marrow of the femur arises from a different germ layer than the bone marrow of the jaw bones; femurs are formed through endochondral ossification while jaw bones are formed through intramembranous ossification. Site specificity may play a role in facilitating bone regeneration, indicating that M-MSCs may be the more suitable MSC option in treating orofacial defects. The promising outcome of MSCs from the mandible will confirm that the M-MSCs are clinically relevant stem cell source in regenerating orofacial bone defect using tissue engineering strategy. It may be beneficial to match the MSCs’ origin with the region that requires regeneration and repair to maximize MSCs’ osteogenic potential.

This study compares the differentiation and bone regeneration potentials between two bone marrow derived MSCs, F-MSCs, and M-MSCs that have been acquired using our methodology to isolate stem cells from rat’s orofacial region. A comparison of the M-MSCs with F-MSCs of the same rat will provide insight into clinical use for bone regeneration, particularly of orofacial bone. In order to correctly conclude that the harvested M-MSCs are true MSCs, basic characteristics of the MSCs, including colony formation, specific surface markers, and differentiation capabilities, will be examined. From that point, the growth of F-MSCs and M-MSCs will be compared. Most relevantly, in vitro and in vivo bone mineralization will be analyzed for MSCs acquired from each of the two source sites.

## Materials and methods

### Isolation of M-MSCs and F-MSCs

**Figure 1.**
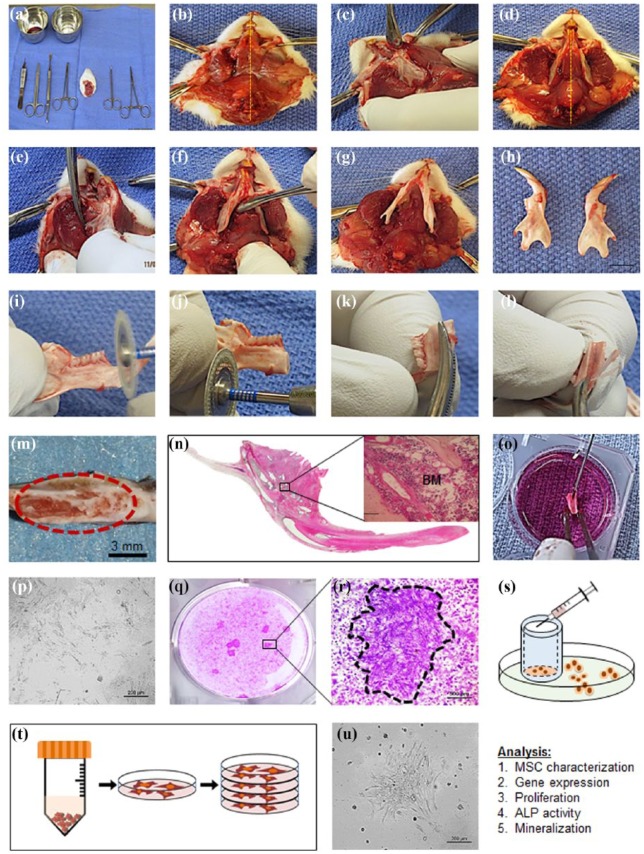
Cell isolation method from Sprague–Dawley rat mandible. After sacrifice, the head was removed along the cervical vertebrae region (a), vertical incision was made along the ventral midline to expose the maxillofacial muscular tissue (b); muscular and connective tissues were dissociated from the lateral sides of the mandible complex using a mucoperiosteal elevator (c and d); the condyle was detached from the glenoid fossa by cutting the temporal mandibular joint disk (e); surrounding ligaments were removed on each side (f); muscular and connective tissues from the medial sides of the mandible were dissociated to facilitate segmentation and extraction of the whole mandible (g); the mandible was divided into two halves by cutting the symphysis, which is located between the lower incisors, with surgical scissors (h); the central part of the mandible was segmented from the mesial and distal of the molar area with a diamond-coated cutting disk (i and j); each mandible was slightly cut on one side and then cracked along the ventral edge to expose the marrow chambers (k, l, m and n); the bone marrow was flushed directly into a 35 mm culture dish with growth media (o); isolated bone marrow was cultured inside the incubator at 37°C and 5% CO_2_, and the media were replaced every 3 days (p); once adherent cells formed colonies on the bottom of the dish and became confluent, colonies were successfully isolated by colony picking method for further expansion (q, r and s); the isolated cells were expanded and cultured inside the incubator at 37°C and 5% CO^2^, and the media were replaced every 3 days (t and u).

All animal studies were followed the guidelines for Institutional Animal Care and Use Committee (ICAUC) at the University of North Carolina at Chapel Hill (approved protocol number 15-273). After Sprague–Dawley (SD) rat (12 weeks) was sacrificed, the head was removed along the cervical vertebrae region. The superficial area was disinfected, and then, a vertical incision was made along the ventral midline to expose the maxillofacial muscular tissue. The muscular and connective tissues were dissociated from the lateral sides of the mandible complex using a mucoperiosteal elevator. The condyle was detached from the glenoid fossa by cutting the temporal mandibular joint (TMJ) disk and the surrounding ligaments on each side. The muscular and connective tissues from the medial sides of the mandible were also dissociated to allow easy segmentation and extraction of the whole mandible. The mandible was divided into halves by cutting the symphysis, which is located between the lower incisors, with surgical scissors. The separated mandible was sterilized with 70% ethanol and iodine solution. After drying, the central part of the mandible was segmented from the mesial and distal of the molar area with a diamond-coated cutting disk. Each mandible was slightly cut on one side and then cracked along the ventral edge to expose the marrow chambers. The bone marrow was flushed directly into a 35 mm culture dish with growth media (Dulbecco’s Modified Eagle Medium (DMEM); Thermo Fisher Scientific Inc., Rockford, IL, USA) supplemented with 10% fetal bovine serum (FBS; Sigma), 1% penicillin and streptomycin (P/S; Thermo Fisher Scientific Inc.), and 250 µL of GlutaMax^®^ (Thermo Fisher Scientific Inc.). The isolated bone marrow was cultured inside the incubator at 37°C and 5% CO_2_, and the media were replaced every 3 days. Once adherent cells formed colonies on the bottom of the dish and became confluent, colonies were isolated by colony picking method for further expansion (Figure 1).

The F-MSCs from the femurs of the same rat were isolated and cultured at the same time with M-MSCs. Femurs were removed and surrounding soft tissues were cleaned. Both ends of the femur were cut to expose bone marrow. The bone marrow was transferred to a 1.5 mL Eppendorf tube and isolated by flushing with culture media. Then, bone marrow was further expanded under growth media, following the same protocol as that of M-MSC expansion and culturing. Cultures obtained from each rat were processed separately.

### Surface marker characterization by flow cytometry and immunostaining

For flow cytometric analysis, M-MSCs and F-MSCs at passage 5 were trypsinized, counted, suspended in phosphate buffered saline (PBS) containing 5% bovine serum albumin (BSA) (5 × 10^5^ cells/30 μL), and then incubated with fluorescein-conjugated antibodies specific for CD44, CD73, CD90, and CD105 (BD Biosciences, USA) at 4°C for 30 min. Negative control cells were stained with an each corresponding fluorochrome-conjugated mouse IgG1 isotype (BD Biosciences). Expression of the MSC surface markers was evaluated with a FACSCalibur flow cytometer (BD Biosciences, San Jose, CA, USA) using CellQuest Pro (BD Biosciences) software.

The MSCs were plated onto six-well plates and allowed to attach for 24 h. Cells were then fixed with cold methanol for 5 min. Following treatments of 1% Triton X-100 in PBS for 30 min and 3% H_2_O_2_ for 40 min, the cells were incubated with primary antibodies, anti-mouse CD44 (BD Biosciences) and anti-mouse CD90 (BD Biosciences) diluted at 1:50, at 4°C overnight. After vigorous washing, cells were incubated at room temperature (RT) for 30 min with Texas Red and fluorescein isothiocyanate (FITC)-conjugated secondary antibodies (BD Biosciences) diluted at 1:300. After washing with PBS, cells were treated with 4′,6-diamidino-2-phenylindole (DAPI) for nuclear staining. The image of the marker expressions was captured using Nikon Eclipse Ti-U inverted fluorescence microscope (Nikon Instruments Inc., Melville, NY, USA).

### Multi-linage differentiation

To evaluate their osteogenic capacity, fifth passage cells were treated with osteogenic media for 3 weeks; media were replaced every 3 days. The osteogenic media were composed of culture media supplemented with 10 mM *β*-glycerophosphate (Sigma-Aldrich, St Louis, MO, USA), and 0.2 mM ascorbic acid (Sigma-Aldrich), and 10^−4^ mM dexamethasone (Sigma-Aldrich).

To induce chondrogenic differentiation, fifth passage cells were transferred into 15 mL polypropylene tubes and centrifuged at 1000 r/min for 5 min to form a pellet at the bottom of the tube. Then, the pellet was treated with chondrogenic media for 3 weeks. Fresh media were supplied every 3 days. Chondrogenic media consisted of low-glucose DMEM (Life Technologies, Grand Island, NY, USA) supplemented with 10^−4^ mM dexamethasone, 50 μg/mL ascorbic acid, 100 μg/mL sodium pyruvate (Sigma-Aldrich), 40 μg/mL proline (Sigma-Aldrich), 10 ng/mL transforming growth factor beta 1 (TGF-*β*1), and 50 mg/mL insulin–transferring–selenium (ITS) premix (Becton Dickinson; 6.25 g/mL insulin, 6.25 g/mL transferrin, 6.25 ng/mL selenius acid, 1.25 mg/mL BSA, and 5.35 mg/mL linoleic acid). Cell pellets were imbedded in optimal cutting temperature (Thermo Fisher Scientific Inc.) and frozen at −80°C. The frozen block was sectioned into 5 μm slices using Crytome (Leica Biosystems Inc., Buffalo Grove, IL, USA), stained with safranin O, and counterstained with fast green.

To induce adipogenic differentiation, fifth passage cells were treated with adipogenic medium for 3 weeks. The media were replaced twice per week. Adipogenic medium consisted of Dulbecco’s Modified Eagle’s Medium (DMEM) supplemented with 0.5 mM 3-isobutyl-1-methylxanthine (IBMX; Sigma-Aldrich), 1 M hydrocortisone (Sigma-Aldrich), 0.1 mM indomethacin (Sigma-Aldrich), and 10% rabbit serum (Sigma-Aldrich). Cells were fixed with 10% formalin and stained with Oil Red O to detect fat droplets.

### Colony forming unit assay

The colony forming unit (CFU) assay was performed using both whole bone marrow and single cell suspension. For the whole bone marrow CFU assay, isolated BMs from each femur and mandible were suspended in 2 mL of media and then plated on 35 mm dishes. For the single-cell CFU assay, cells (passage 5) were suspended in growth medium at a concentration of 100 cells/mL. Two milliliter of the cell suspension was plated in a 35 mm culture dish and the fresh media were supplied every 3 days. On the 15th day, cultures were fixed with 10% neutral formalin and stained with crystal violet. After complete dry, colonies larger than 1000 µm in diameter were counted from the image acquired with Nikon imaging system and DP70 color digital camera equipped with color image software (DP11; Olympus USA, Center Valley, PA, USA). Each assay was examined in triplicate from three different rats.

### Osteogenic gene expression

Real-time polymerase chain reaction (PCR) for early osteogenic gene expression of M-MSCs and F-MSCs was performed after culturing the MSCs under growth and osteogenic media for 2 and 5 days, respectively. The control groups for both M-MSCs and F-MSCs were cultured in growth media. Real-time PCR was performed using 7200 Fast Real-Time PCR System (Applied Biosystems, Bedford, MA, USA) to determine mRNA expression of each osteogenic specific gene during osteogenic differentiation. Primers used for the real-time PCR are listed in Table 1. RNAs were isolated using RNA isolation kit (Qiagen, Valencia, CA, USA), and then cDNA was synthesized using iScript™ cDNA Synthesis Kit (Bio-Rad, Hercules, CA, USA) and the company instructions. All PCR data were normalized with glyceraldehyde-3-phosphate dehydrogenase (GAPDH) expression

**Table 1.** Primers used in real-time polymerase chain reaction (PCR) performed to measure osteogenic gene expression.

Target	Primer sequence (5′–3′)	Size (bp)
ALP	F: AACCCAGACACAAGCATTCC R: GCCTTTGAGGTTTTTGGTCA	200
BSP	F: CCGGCCACGCTACTTTCTT R: TGGACTGGAAACCGTTTCAGA	66
OCN	F: CTGACCTCACAGATGCCAA R: GGTCTGATAGTCTGTCACAA	185
GAPDH	F: TGAGGTGACCGCATCTTCTTG R: TGGTAACCAGGCGTCCGATA	101

ALP: alkaline phosphatase; BSP: bone sialoprotein; OCN: osteocalcin; GAPDH: glyceraldehyde-3-phosphate dehydrogenase.

### Proliferation assay

The proliferative potentials of M-MSCs and F-MSCs were measured using 3-(4,5-dimethylthiazol-2-yl)-5-(3 carboxymethoxyphenyl)-2-(4-sulfophenyl)-2H-tetrazolium (MTS) assay. Cells (passage 5) were seeded at a density of 20,000 cells per 35 mm plate and cultured with the growth media. Each sample was triplicated. After 1, 3, 5, and 7 days of culture, the medium was removed, the cells were washed with PBS, and 540 µL of MTS solution (500 µL of DMEM + 40 µL of MTS solution) was added to each well. Plates without cells were used as blanks. After incubation at 37°C for 1 h, 100 µL of the reacted MTS solution was transferred to a 96-well plate, and the absorbance was measured using a plate reader (Molecular Devices, LLC, San Jose, CA, USA) at wavelength of 490 nm. The absorbance values for each time point for M-MSCs and F-MSCs were then used to calculate doubling time at website (http://www.doubling-time.com/compute.php).

### Alkaline phosphatase activity

M-MSCs and F-MSCs (10^5^ cells per well) in 12-well plates were cultured for 4, 7, and 11 days in growth medium (α-MEM, 10% FBS, 1% penicillin and streptomycin, and 1× GlutaMax) with or without osteogenic inducing factors (10 mM *β*-glycerophosphate, 0.2 mM ascorbic acid, and 10^−4^ mM dexamethasone). The media replaced every 2 days. The cells were harvested on days 4, 7, and 11 and centrifuged at 3000 r/min for 10 min. Then, cell pellets were lysed at 4°C using 1% Triton-X 100 in 1× PBS with occasional voltexing for 20 min. Supernatants were collected to calculate the total protein quantity. Alkaline phosphatase (ALP) was calculated using colorimetric assay (#ab83369; Abcam, Cambridge, UK) following the manufacturer’s instructions. To quantify ALP in each sample, the colorimetric reagent *p*-nitrophenyl phosphate (pNPP) and its hydrolyzable phosphate were used as the standard. The reaction was stopped using 0.1 N NaOH when measuring absorbance at 405 nm.

### In vitro mineral nodule formation

To measure and compare bone mineralization capabilities of the M-MSCs and F-MSCs, 20,000 cells were seeded per well in 12-well culture dishes. Fresh osteogenic media (growth media containing 10 mM *β*-glycerophosphate, 0.2 mM ascorbic acid, and 0.1 mM dexamethasone) were supplied every 3 days for 28 days. The cells were harvested on days 10, 14, 21, and 28 to be fixed in 95% ethanol for 30 min at room temperature. The fixed cells were washed with PBS and stained with 1% Alizarin Red Solution (pH 4.2) for 10 min at room temperature. Quantitative analysis was performed by elution with 10% (w/v) cetylpyridium chloride for 10 min at room temperature, and the optical density (OD) was measured at 570 nm.

### In vivo bone formation using rat critical sized defect

All animal studies followed the guidelines for ICAUC at the University of North Carolina at Chapel Hill (approved protocol number 15-273). Total 5 × 106 rat mesenchymal stem cells (rMSCs) differentiated in osteogenic media for 14 days were seeded on Gelfoam^®^ (10 mm in diameter × 4 mm in thickness). Three implantation groups (each group has four rats, n = 4) were used (Gelfoam only, Gelfoam seeded with F-MSCs, and Gelfoam seeded with M-MSCs). A critical sized defect (CSD; 8 mm in diameter) was created at the center of calvaria of SD rats after they received peritoneal injection of Ketamine HCl (10 mg/kg; Putney Inc., Portland, ME, USA). The detailed surgical procedure has been well described in a previous study.^15^ Each rat was euthanized by CO_2_ overdose and its calvarium was harvested 8 weeks post-implantation.

### MicroCT analysis

The calvaria explants were scanned with Skyscan microCT (Skyscan 1076; Skyscan, Aartselaar, Belgium) at 40 kV, 1000 mA with a 720 ms integration time. Three-dimensional images were reconstructed using ITK-SNAP software. Following the reconstruction, newly formed bone in CSD was measured using Geomagic Design X™ (3D Systems Inc., Rock Hill, SC, USA) software for three animals in each group. Animals that received implantation of Gelfoam without cells served as the control. The bone volume was represented in cubic millimeter.

### Histology and new bone formation measurement

After 8 weeks of implantation, Calvaria were harvested, immediately fixed in 10% neutral formalin solution, and decalcified in 14% ethylenediaminetetraacetic acid (EDTA) in saline (pH 7.4) for 21 days. Then, tissues were dehydrated, embedded in paraffin, and sectioned to expose mid-sagittal view of the calvaria. Hematoxylin and eosin and Masson trichrome staining were performed to assess the new bone formation (NBF). H&E staining was done in University of North Carolina animal pathological core and Masson trichrome staining was performed using Trichrome Stain kit (HT-15; Sigma-Aldrich) and following the company instruction. Color images of the histologic sections were acquired under 10× magnification using Nikon Eclipse Ti-U camera with automatic stage (Nikon Instruments Inc.), and image analysis was performed by measuring in pixels using Image J software (U.S. National Institutes of Health, Bethesda, MD, USA). The newly formed bone area (B.Ar.) was calculated in percentage by dividing by the total defect area (T.Ar.).

### Statistics

All results were represented as mean ± standard deviation. Significant differences among groups were defined using analysis of variance (ANOVA). The differences were considered significant (*) when the *p* value was smaller than 0.05.

## Results

### Isolation of MSCs from rat mandible

Before isolating M-MSCs from rat mandible bone marrow, the location of each bone marrow cavity was confirmed by H&E staining. The detailed MSC isolation method is described in a step-by-step manner in Figure 1. M-MSCs were successfully isolated from mandible and expanded for further characterization and assessment of osteogenic potential. F-MSCs from the femur of the same rats were also isolated and expanded to be used for comparison. Out of 20 SD rats used, M-MSCs were successfully isolated and expanded from 16 rats (80%) while F-MSCs were successfully isolated and expanded from 19 rats (95%). All experiments in this study were repeated three times with independently isolated MSCs, and the results were averaged.

### Characterization of the M-MSCs

**Figure 2.**
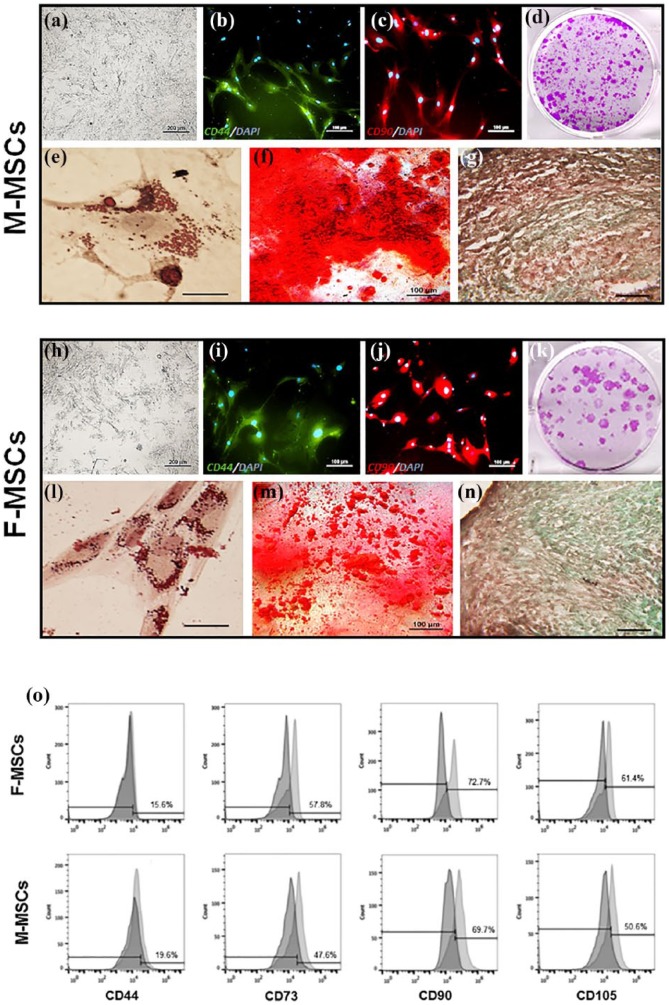
Characterization of M-MSCs and F-MSCs. F-MSCs were used as control. MSCs were characterized for plastic adhesion (a and h), CD44 antibodies (b and i), CD90 antibodies (c and j), colony forming capability (d and k), adiopogenicity (e and l), mineral nodules formation (f and m), and chondrogenesis (g and n) in vitro. Both MSCs exhibited elongated fibroblastic morphology and expressed the expected MSC-specific CD markers and tri-lineage differentiation potentials. Flow cytometric analysis of M-MSCs and F-MSCs. M-MSCs express similar levels of MSC-associated markers, CD44, CD73, CD90, and CD105, as F-MSCs (o).

After 24 h, the M-MSCs adhered to the plastic culture dish and exhibited a typical elongated fibroblastic morphology (Figure 2(a)) similar to that of F-MSCs (Figure 2(h)). At passage 5, both M-MSCs and F-MSCs clearly expressed MSC-specific markers, CD44 (Figure 2(b) and (i)) and CD90 (Figure 2(c) and (j)), as observed by immunohistochemical staining. In addition, both MSCs were negative for CD14, CD45, and CD34 (data not shown). For the tri-lineage differentiation potential, M-MSCs and F-MSCs were able to successfully differentiate into the osteogenic, chondrogenic, and adipogenic lineages. Although the levels of each differentiation were not quantified for comparison, both MSCs formed crude mineral nodules for osteogenic differentiation (Figure 2(f) and (m)), glycosaminoglycan for chondrogenic differentiation (Figure 2(g) and (n)), and fat droplets for adipogenic differentiation (Figure 2(e) and (l)).

From flow cytometric analysis (Figure 2(o)), both M-MSCs and F-MSCs were positive for CD44 (15.6% for M-MSCs and 19.6% for F-MSCs), CD73 (57.8% for M-MSCs and 47.6% for F-MSCs), CD90 (72.7% for M-MSCs and 69.7% for F-MSCs), and CD105 (61.4% for M-MSCs and 50.6% for F-MSCs), while negative for CD45 and CD34 (data not shown). M-MSCs showed higher expression level on CD73, CD90 and CD105 and lower on CD44 than F-MSCs.

### Proliferative potential of the M-MSCs

**Figure 3.**
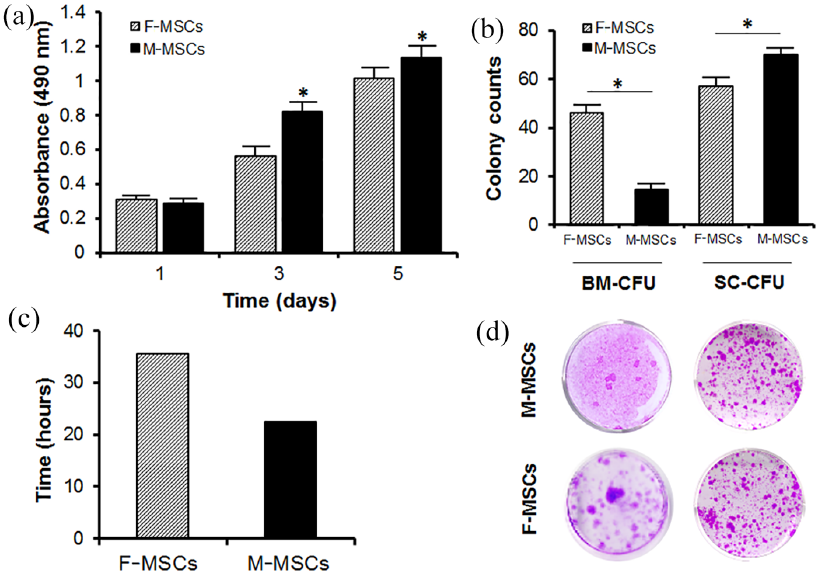
MTS proliferation assays were performed on days 1, 3, and 5 in 12-well plates with n = 5 measurements from six independent samples per group. Doubling time (DT) was calculated using equation, DT = Ln(2)/growth rate (amount = 0.2119 × e0.0096 × time). At t = 0 h, cell concentration was 0.2119, and 0.2669 when t = 24 h. (a) Comparison of colony forming unit between M-MSCs and F-MSCs. (b) Comparison of colony counts between M-MSCs and F-MSCs. (c) Comparison of doubling time between M-MSCs and F-MSCs. (d) Colony forming assay results for bone marrow and single cell cultures. M-MSCs demonstrated significantly higher proliferation rates and lower doubling time compared to F-MSCs.

Proliferations of M-MSCs and F-MSCs were examined by measuring the mean values of OD at days 1, 3, and 5 using MTS assay (Figure 3(a)). Population of M-MSCs and F-MSCs increased until day 5; both MSCs became confluent afterward. In comparison to the F-MSCs, the M-MSCs exhibited significantly higher (*p < 0.05) proliferation rates, indicated by their OD, 0.82 ± 0.26 and 1.13 ± 0.41 on days 3 and 5, respectively. Overall, M-MSCs demonstrated a lower doubling time (22.6 ± 2.22 h) than F-MSCs (35 ± 3.19 h) (Figure 3(c)).

### CFU assay

To test colony forming capability, CFU assays were performed using both whole bone marrow and single cells from the mandible and femur. The amount of bone marrow harvested from the mandible was less than that from femur. For the whole bone marrow CFU assay, BM from mandible yielded 15 ± 3 colonies, while bone marrow from femur yielded 46 ± 4 colonies after 14 days of culturing in growth media (Figure 3(b) and (d)). While M-MSCs formed a significantly less number of colonies than F-MSCs in the whole bone marrow CFU assay, M-MSCs generated a significantly higher number of colonies (70 ± 3 colonies) than F-MSCs (57 ± 4 colonies) in single-cell CFU assay.

### Osteogenic gene expression

**Figure 4.**
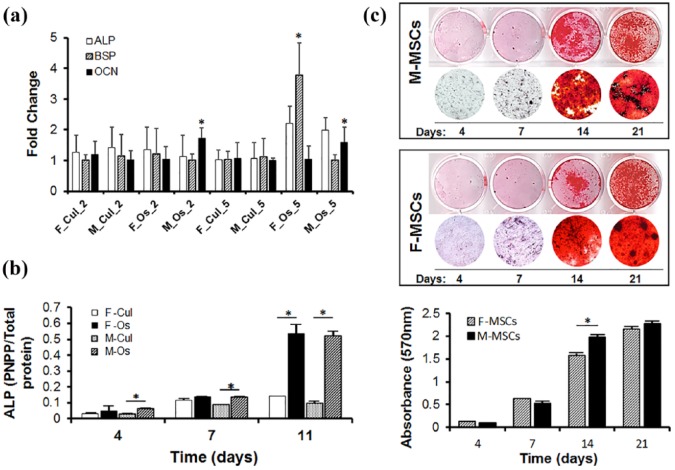
Assessment of osteogenic differentiation of M-MSCs and F-MSCs. The osteogenic gene expressions of M-MSCs and F-MSCs were evaluated by culturing both M-MSCs and F-MSCs in growth and osteogenic media, then using real-time PCR analysis to detect ALP, BSP, and OCN genes. Real-time PCR data were normalized with GAPDH expression (n = 3 per group; *p < 0.05) (a). Alkaline phosphatase (ALP) activity by M-MSCs and F-MSCs after being cultured in growth and osteogenic media for 4, 7, and 11 days, respectively. The symbol (*) represents significance in ALP activity (b). Mineral formation was detected by Alizarin Red staining (c) after culturing M-MSCs and F-MSCs with under osteogenic media; growth media served as the control. Microscope images confirmed the MSCs’ ability to mineralize at 10, 17, and 28 days of osteogenic differentiation. Alizarin Red S stained particles were quantified by the CPC extraction method, with the absorbance of the extracted solution being measured at 570 nm (n = 5, *p < 0.05) (c). ALP expression was significantly higher in M-MSCs and F-MSCs cultured in osteogenic media. M-MSCs exhibited lower BSP expression level and smaller increase in OCN level than M-MSCs in osteogenic media. Both M-MSCs and F-MSCs successfully formed mineral nodules; mineralization of F-MSCs was significantly higher than that of M-MSCs in later time period.

Real-time PCR was used to determine differences in osteogenic gene expression level between M-MSCs and F-MSCs during induction with osteogenic media. Expression of ALP gene was significantly higher in both M-MSCs (1.73 folds) and F-MSCs (1.05 folds) at day 5 in osteogenic media culture compared to the levels at day 2 (Figure 4(a)). Expression of bone sialoprotein (BSP) was significantly higher in F-MSCs (3.78 folds, *p* < 0.05) than in M-MSCs (1.01 folds) cultured in osteogenic media for 5 days. M-MSCs in osteogenic media also showed a smaller increase of osteocalcin (OCN) gene expression (1.73 ± 0.34 fold on day 2 to 1.59 ± 0.48 fold on day 5) than F-MSCs in either growth or osteogenic media. While F-MSCs showed higher expressions of ALP and BSP genes under osteogenic media on day 5, OCN gene expression by M-MSCs was higher in osteogenic media on days 2 and 5 (Figure 4(a)).

### ALP activity

The results of ALP activity assay of M-MSCs and F-MSCs in growth and osteogenic media are shown in Figure 4(b). Overall, M-MSCs’ level of ALP activity comes close to that of F-MSCs on days 4, 7, and 11 of differentiation in osteogenic media. F-MSCs showed slightly higher ALP levels than M-MSCs on days 7 and 14 in culture media. Overall, these results suggested that both M-MSCs and F-MSCs have a similar potential for ALP activity during culture and osteogenic condition.

### In vitro mineralization

We investigated whether M-MSCs could be effectively differentiated by osteogenic media like F-MSCs. The mineral nodules of M-MSCs and F-MSCs were visualized by Alizarin Red S (ARS) staining and quantified by cetylpyridinium chloride (CPC) extraction after 4, 7, 14, and 21 days of osteogenic differentiation. We observed that mineral nodules formed by M-MSCs and F-MSCs started to appear on 14 days of osteogenic differentiation and increased over time up to 21 days (Figure 4(c)). Mineralization of F-MSCs was significantly higher (1.98 ± 0.05) than of M-MSCs (1.57 ± 0.05) on day 14, as indicated by CPC quantification and ARS staining. Mineralization was not induced in either M-MSCs or F-MSCs under growth media (data not shown).

### In vivo bone formation

**Figure 5.**
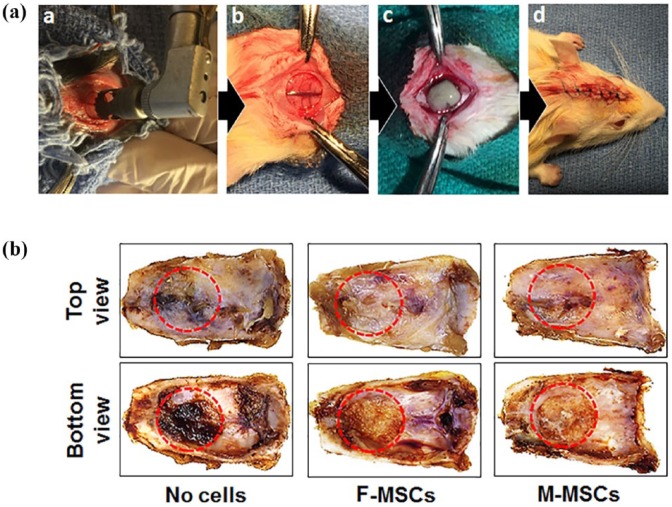
Implantation of MSCs seeded Gelfoam^®^ in the rat calvarial critical sized defect (a). Growth images of calvarial explants after 8 weeks of implantation with Gelfoam only, Gelfoam with F-MSCs, and Gelfoam with M-MSCs (b). Dotted lines outline the original critical sized defects (8 mm in diameter).

Gelfoam incorporating M-MSCs and F-MSCs were implanted into rat calvarial CSDs to examine bone regeneration (Figure 5(a)). After 8 weeks of-implantation, the animals were sacrificed and their calvaria were carefully removed for further analysis (Figure 5(b)). To quantify NBF in CSD, microCT and histological (H&E and Masson’s trichrome staining) analyses were used after fixation.

**Figure 6.**
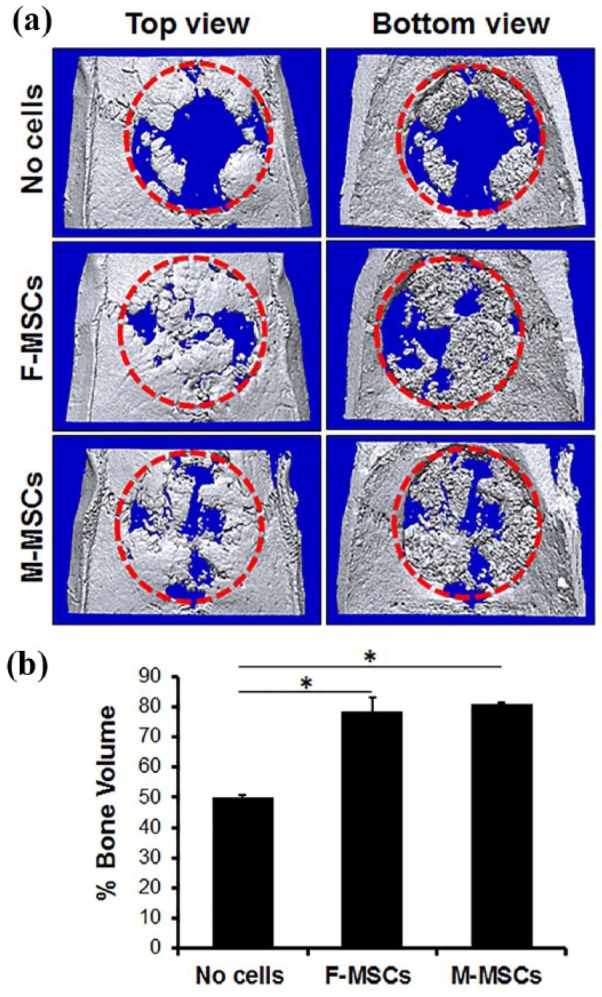
Comparison of calvarial bone regeneration on rat CSD by MicroCT analysis. M-MSCs and F-MSCs were carried by Gelfoam sponge in the CSD, and bone regeneration was observed after 8 weeks. Top and bottom views of 3D MicroCT images of CSD (red dotted circle) post-implantation of Gelfoam only, Gelfoam with M-MSCs, and Gelfoam with F-MSCs (a). Quantitative measurement of the defect filling in percentage, *p < 0.05 (b). Both M-MSCs (80.88% ± 0.68%) and F-MSCs (78.73% ± 4.32%) regenerated significant volumes of new bone in comparison to Gelfoam only group (49.87% ± 0.94%).

MicroCT images indicated the CSD with Gelfoam only group remained largely empty at the center of the defect with some mineralized tissue formation confined mostly to the defect edges (Figures 6(a)). In contrast, defects implanted with Gelfoam with M-MSCs or F-MSCs exhibited coverage with mineralized tissue throughout the defect, confirmed by high bone volume (Figure 6(a)). Although there were some central regions where no bone formation was evident, the edges of the regenerated calvarial defect appeared continuous with the surrounding bone. In addition, bridging occurred across Gelfoam, where newly regenerated bone connects one end of the defect to the other end. Newly formed bone volume was measured to be 80.88% ± 0.68% for the Gelfoam with M-MSCs group, 78.73% ± 4.32% for the Gelfoam with F-MSCs group, and 49.87% ± 0.94% for the Gelfoam only group (Figure 6(b)). As shown by the higher volume in Figure 6(b), there was greater formation of new bone observed in both the Gelfoam with M-MSCs and Gelfoam with F-MSCs groups than the Gelfoam only group. Overall, greater bone regeneration in the defect site occurred when using Gelfoam with M-MSCs and Gelfoam with F-MSCs (Figure 6). In addition, there was no significant difference in new bone volume between Gelfoam with M-MSCs and Gelfoam with F-MSCs group (*p* > 0.05).

**Figure 7.**
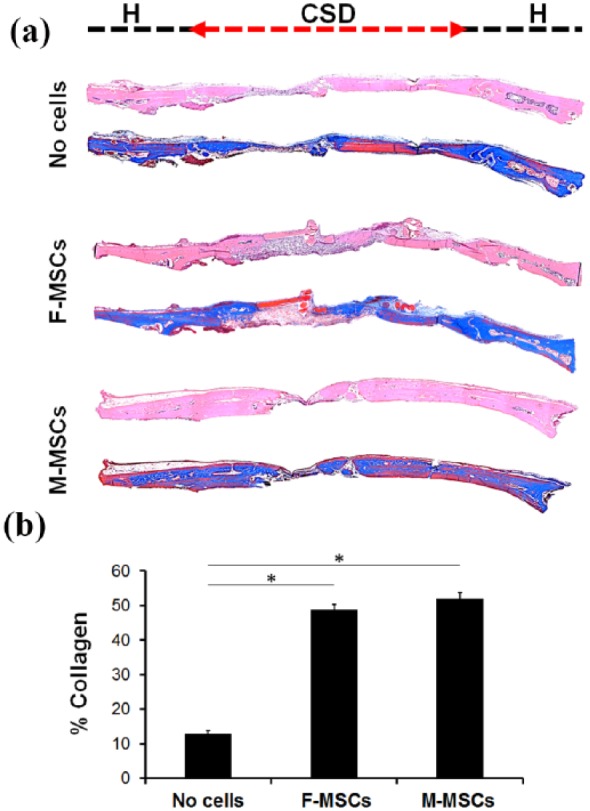
Histological analysis (H&E and Masson’s trichrome staining) of the newly formed bone in the critical sized defect by the M-MSCs and F-MSCs. The newly formed bone in M-MSCs group contained similar collagen positive bone matrix (blue) as F-MSCs group. CSD indicates the defect area (a). Quantitative measurement of collagen in the defect in percentage, *p < 0.05 (b). While Gelfoam only group resulted in minimal new bone formation (12.9% ± 0.8%), M-MSCs (51.99% ± 1.85%) and F-MSCs (48.79% ± 1.56%) demonstrated comparable and successful new bone formation.

H&E histological section revealed that defects implanted with Gelfoam only were bridged with a thin layer of fibrous connective tissues, rather than with host bone tissue (Figure 7(a)). Gelfoam with M-MSCs or F-MSCs regenerated thick mineralized tissue in the CSD (Figure 7(a)) unlike the Gelfoam only group. Further analysis using sections stained with Masson’s trichrome showed amounts of newly formed collagen fibers, which is the platform for the mineralization. The newly formed bone was well integrated at the interface between the host bone and implants in both M-MSCs and F-MSCs groups. In addition, no NBF was observed in the defect only group; instead, fibrous tissue formation was detected in the defect area. The quantitative measurements of NBF demonstrated 51.99% ± 1.85% bone regeneration for the Gelfoam with M-MSCs, 48.79% ± 1.56% for the Gelfoam with F-MSCs, and 12.9% ± 0.8% for the Gelfoam only group (Figure 7(b)). Overall, M-MSCs showed a high potential to regenerate bone, comparable to F-MSCs’ potential.

## Discussion

Cell source has emerged as a leading factor in tissue engineering application to repair large bone defect. In the present, MSCs are the most prominent adult stem cell source and have been used in numerous studies for bone tissue engineering. Their specific role is to facilitate tissue engineered bone regeneration by forming mineralized extracellular matrix (ECM) in the scaffold material. MSC-like cells have been isolated from tissues in the dental and craniofacial region such as dental pulp, periodontal ligament, and apical papillae; various degrees of bone regenerations have already been reported for these cells.^14,16^ The major limitations of those dental stem cells are that tooth have to be devitalized or extracted to isolate MSC-like cells. Compared with those dental stem cells, M-MSCs can be isolated in a less invasive manner than the dental stem cells. Furthermore, as our results have indicated in this study, only small amount of the isolated bone marrow from the mandible is necessary to expand in vitro and their osteogenic potential appears to be similar to F-MSCs.

Another rationale of using M-MSC for craniofacial bone regeneration is the possibility to enhance bone regeneration based on their site-specific properties. Since bone marrow is of mesodermal origin, we believe that the MSCs isolated from bone marrow in the mandible can also be recognized as a promising candidate for craniofacial bone regeneration due to their shared bone marrow origin. Craniofacial bone is known to have originated from the ectomesenchymal lineage, which closely interacts with the nervous system.^17^ However, it is not clearly known how the bone marrow–derived mesenchymal stem cells (MSCs) of ectomesenchymal lineage differ from that of mesenchymal lineage. The site-specific characteristic of the M-MSCs from the mandible might have contributed to the improved outcome compared to the results when F-MSCs from the femur were used in bone regeneration. According to the study done by Akintoye and colleagues,^18,19^ MSCs isolated from orofacial region (maxilla and mandible) and axial bone (iliac crest) indicated the existence of skeletal site-specific properties in terms of different embryological origins. However, what we know is that the majority of the therapeutic applications involved long bone marrow–derived MSCs, and that there is little to no studies comparing the osteogenic differentiation potentials between the rMSCs that were isolated from either mandibles or femurs. In addition, the fundamental mechanisms and clinical implications in bone regeneration are still not clearly understood and require further extensive studies. Therefore, assessing site-specific differences between M-MSCs and F-MSCs is of interest.

In our study, we investigated the feasibility of MSCs isolated from two different origins, mandible and femur, to serve as a cell source for bone regeneration, specifically in repairing calvarial CSD. M-MSCs used in our study were isolated from the bone marrow of rat mandible by selecting for CFUs, a common isolation method for F-MSCs. We confirmed that the isolated M-MSCs could differentiate into any of the tri-lineages, which include adipogenic, chondrogenic, and osteogenic lineages, just as F-MSCs could. The short-term proliferative potential of M-MSCs was quite comparable to that of F-MSCs and the overall osteogenic potential of M-MSCs was nearly the same as F-MSCs. Such results indicate that M-MSCs are almost identical to F-MSCs and may represent a prominent MSC source for craniofacial bone regeneration. However, the application and potential of the M-MSCs in long bone regeneration is still not clear, and thus, further studies are necessary to clarify the site-specific effect of MSCs based on the tissue origin.

Bone marrow–derived MSCs have been most widely used for bone tissue regeneration due to their immunomodulatory and trophic effects, which enable allogeneic application.^19,20^ Allogeneic MSCs are an attractive cell resource in clinical stem cell therapy as they are readily available with significantly reduced cell preparation time. Although our study utilized allogeneic MSCs mainly due to the limited amount of available tissues and limited size of the animal, autologous MSCs are still the preferred cell source in real clinical setting.

One of the concerns with using MSCs isolated from mandible is the high risk of microorganism contamination from oral cavity. Such contamination will have a critical effect on the MSCs culture during expansion of the cells. The isolated cells were thoroughly sterilized with iodine and 1% antibiotic solution to prevent any contamination. Out of total 20 rats, we could successfully isolate and culture M-MSCs from 16 rats.

In our study, the initial yield of the M-MSCs from mandibular was much lower than that of femur. Commonly, due to the unique anatomical feature of the mandible that limits the amount of acquirable MSCs, the bone marrow in mandible has been thought as less attractive source of MSCs for bone regeneration. In rat model, the amount of bone marrow in rat mandible is quite limited compare to that in long bone. However, we proved that it is possible to isolate sufficient amount of MSCs from the BM in the rat mandible and the MSCs could expand enough to be considered a source of M-MSCs for bone tissue engineering therapies. The difference in the amount of bone marrow had a direct influence over the colony forming result. Therefore, we performed single-cell CFU assay to evaluate the sole colony forming potential of the rMSCs from each origin of tissues and confirmed the comparable colony forming potential of M-MSCs. Interestingly, the proliferation rate of M-MSCs is higher than that of F-MSCs during 7 days of culture by representing shorter doubling time. This suggests that the mandible may contain a more reliable and consistent source of MSCs compared with the femurs.

In human case, the mandible is an adequate donor site, where a sufficient amount of bone marrow (10 mL) can be harvested by surgical aspiration.^20^ Furthermore, the bone formation using the M-MSCs is remarkably consistent in healthy patients, suggesting that marrow aspirates could be readily obtained from most patients.^21^ Thus, studying those rat M-MSCs are predictive of human responses in bone regeneration. Several studies have demonstrated in vivo bone regeneration of orofacial MSCs in ectopic sites, but no one had evaluated the cells in orthotopic sites using rat CSD model. The success of M-MSCs in regenerating bone could not only increase the amount of resources as the mandible is more accessible, especially for those who focus on orofacial regions like dentists, but also eliminate the need for additional personnel as there is no need to harvest MSCs from non-orofacial regions, such as the femur.

Although our result demonstrated that M-MSCs may have useful and promising applications in bone regeneration, further studies are needed to optimize the osteogenic potential of M-MSCs in the bone scaffolding environment and to determine whether M-MSCs have the capacity to repair large bone defects. While Gelfoam, a collagen-based natural material, was used to eliminate biomaterial stimulation and focus on MSCs’ effect on osteogenesis in this study, to repair the large bone defect such as CSD, bone scaffold is necessary. The potential of orofacial derived MSCs in large bone defect regeneration is yet to be explored. Together with bone scaffold, M-MSCs are expected to be able to serve as a new therapeutic cell source for bone regeneration using tissue engineering approaches, especially for the orofacial region.

## Conclusion

Our results demonstrated that the M-MSCs isolated from rat mandible are comparable in proliferation and osteogenic differentiation capacities to F-MSCs in vitro. Implantation of M-MSCs into a rat calvarial CSD also yielded a similar degree of local bone formation as F-MSCs in vivo. Overall, this study showed a strong evidence that M-MSCs can be osteogenically differentiated both in vitro and in vivo.

In conclusion, M-MSCs can serve as an effective alternative cell source for the bone tissue engineering and regeneration. Utilizing these M-MSCs, the future study will be directed toward testing the potential of M-MSCs-seeded-bone scaffolds in craniofacial bone regeneration.
